# Case Report: Persistent isolated hyperhomocysteinemia in an adolescent with celiac disease and homozygous *MTHFR* c.665C>T polymorphism: a multifactorial disturbance of one-carbon metabolism

**DOI:** 10.3389/fgene.2026.1912912

**Published:** 2026-08-26

**Authors:** Zainab Al Masseri, Christian R. Marshall, Nicole Martin, Dawn Cordeiro, Laura Guilder, Michal Inbar-Feigenberg

**Affiliations:** 1 Division of Clinical and Metabolic Genetics, Hospital for Sick Children, University of Toronto, Toronto, ON, Canada; 2 Division of Genome Diagnostics, The Hospital for Sick Children, Toronto, ON, Canada; 3 Laboratory Medicine and Pathobiology, University of Toronto, Toronto, ON, Canada

**Keywords:** celiac disease, hyperhomocysteinemia, methylfolate, MTHFR polymorphism, vitamin B12, adolescent

## Abstract

Hyperhomocysteinemia in adolescence typically prompts investigation for classical inborn errors of sulfur amino acid metabolism, including cystathionine β-synthase deficiency and cobalamin-dependent remethylation disorders. However, persistent elevations may also arise from interactions between common genetic polymorphisms and acquired nutritional conditions that alter one-carbon metabolism.

**Case presentation:**

We report an 18-year-old male with type 1 diabetes mellitus, celiac disease on a strict gluten-free diet, congenital unilateral sensorineural hearing loss, and persistent isolated hyperhomocysteinemia. At age 16, he presented with an acute visual field disturbance and right occipital cortical MRI changes suggestive of ischemia. Plasma homocysteine was persistently between 50 and 65 μmol/L.Extensive metabolic and genetic investigations, including targeted gene panels, whole-exome and research whole-genome sequencing, mitochondrial DNA sequencing, mitochondrial complex activities in fibroblasts, and fibroblast complementation, excluded classical homocystinuria and remethylation defects. Sequential trials of pyridoxine, hydroxocobalamin, and high-dose betaine produced modest or transient improvement. Re-analysis of exome data showed homozygosity for the common *MTHFR* c.665C>T (p.Ala222Val) variant. Family testing revealed that his father (TT) and mother (CT) had normal homocysteine (10.9 and 10.4 μmol/L), indicating that *MTHFR* TT alone is insufficient to cause a biochemical phenotype and functions as a susceptibility factor. Combined oral methylfolate (1,000 µg daily) and vitamin B12 (cyanocobalamin 1,000 µg daily) reduced homocysteine from 66 to 24.6 μmol/L in 8 weeks.

**Conclusion:**

This case illustrates multifactorial hyperhomocysteinemia arising from interactions between celiac disease-related micronutrient vulnerability and reduced *MTHFR* activity. Recognition of gene-nutrient interactions is important when classical metabolic disorders are excluded and may guide targeted therapy.

## Introduction

Marked hyperhomocysteinemia in children and adolescents usually raises suspicion for inherited disorders of sulfur amino acid metabolism. The principal genetic causes, such as cystathionine β-synthase (CBS) deficiency and disorders of intracellular cobalamin metabolism, typically present with elevated homocysteine and characteristic biochemical abnormalities involving methionine, cystathionine, or methylmalonic acid (Morris et al., 2017; Froese et al., 2019).

Homocysteine occupies a central position at the intersection of folate, cobalamin, and vitamin B6-dependent pathways within one-carbon metabolism. Consequently, elevations may arise not only from rare enzymatic defects but also from common genetic polymorphisms interacting with nutritional or inflammatory conditions (Froese et al., 2019; Stabler, 2013).

The *MTHFR* c.665C>T (p.Ala222Val) variant is a common polymorphism associated with homocysteine metabolism. Homozygosity reduces enzyme activity and predisposes to elevated homocysteine, particularly when folate status is suboptimal. In populations of European (Caucasian) ancestry, roughly 10%–15% are homozygous (TT) and about one-third are heterozygous (CT), giving a T-allele frequency of approximately 30%–35%. (Graydon et al., 2019; Huang et al., 2018). The variant is currently designated as c.665C>T; its previous designation was c.677C>T. Both names refer to the same variant under different numbering conventions. Celiac disease provides a physiological setting in which micronutrient malabsorption, chronic inflammation, and altered folate and cobalamin status converge to perturb methylation pathways. Even in patients adhering to a strict gluten-free diet, folate and vitamin B12 intake are often suboptimal, and homocysteine concentrations may remain elevated (Saibeni et al., 2005; Hadithi et al., 2009).

We describe an adolescent with persistent isolated hyperhomocysteinemia in whom a comprehensive investigation excluded classical metabolic disorders. This case illustrates how celiac disease and homozygous *MTHFR* c.665C>T polymorphism can interact to produce clinically relevant metabolic abnormalities and highlights the therapeutic potential of targeted cofactor supplementation.

### Clinical history

An 18-year-old male was followed by Metabolic Genetics for persistent isolated hyperhomocysteinemia. His medical history included type 1 diabetes mellitus diagnosed in early childhood, biopsy-confirmed celiac disease diagnosed at age 10 with long-standing adherence to a strict gluten-free diet, congenital left-sided sensorineural hearing loss, and astigmatism.

At age 16, he experienced an acute onset of visual field disturbance accompanied by headache without loss of consciousness. Brain MRI revealed small areas with subtle right occipital cortical T2/FLAIR hyperintensity interpreted as possibly ischemic. Follow-up imaging showed a mild, localized prominence of the sulcal spaces, subtle FLAIR hyperintense signal changes, and localized thinning of the cortical ribbon within the right occipital lobe. During the stroke work-up, plasma total homocysteine was found to be elevated (50–65 μmol/L). The patient had no clinical features of classical homocystinuria: growth and development were normal, there was no Marfanoid habitus, lens dislocation, or history of thromboembolism. Initial metabolic investigations demonstrated normal plasma methionine, cystathionine, and methylmalonic acid consistent with isolated hyperhomocysteinemia. Other metabolic investigations, including acylcarnitine profile, total and free carnitine levels, urine organic acids and urine nitroprusside testing, were reassuring. Initial B12 level was 282 (180–655 pmol/L) and the initial folate level was 574 (182–834 nmol/L).

### Therapeutic trials

A structured series of therapeutic interventions was undertaken. Pyridoxine supplementation produced no significant change in homocysteine levels, excluding vitamin B6-responsive CBS deficiency. Intramuscular hydroxocobalamin was initiated at 5 mg daily and later escalated to 10 mg daily. This resulted in an initial decline in homocysteine to approximately 34 μmol/L, but the effect was not sustained despite markedly supraphysiologic serum vitamin B12 concentrations. Oral betaine was introduced and gradually escalated to 6 g twice daily, yielding only modest and inconsistent biochemical improvement.

Over 3 years, plasma homocysteine remained persistently elevated, typically ranging between 50 and 60 μmol/L. Serial plasma amino acid assays showed intermittent mild elevations in methionine (up to 55 μmol/L, with two values in the high-normal to mildly elevated range of 51–52 μmol/L, reference 13–44 μmol/L) and mostly levels at the normal reference range of 18–39 μmol/L and low or low-normal serine (approximately 69–84 μmol/L, reference 112–216 μmol/L, on multiple occasions), with glycine drifting toward the lower end of normal at times (183 μmol/L at presentation, reference 218–407 μmol/L) but mostly normal and otherwise unremarkable amino acid profiles, a pattern more consistent with altered one-carbon flux than with a discrete inborn error of sulfur amino acid metabolism.

### Clinical course

Neurologically, the patient remained stable without recurrent stroke-like episodes or thromboembolic events. He continued to experience brief focal visual seizures, described as flashing static with preserved awareness, occurring approximately once weekly and lasting 15–30 s, and was treated with Keppra and carbamazepine. Over several visits, both Keppra and carbamazepine were gradually tapered and ultimately discontinued, without any significant change in the frequency or severity of his brief visual auras. A single episode of a left hemifield “crescent-shaped” visual phenomenon lasting 10 min, occurring in the setting of sleep deprivation, dehydration and prolonged fasting, was felt to be more consistent with migraine with visual aura than an epileptic event. He successfully transitioned to adult neurology care, maintained full academic performance at university, and remained fully independent in his daily activities.

### Targeted cofactor therapy

Based on the hypothesis that impaired folate-dependent remethylation was the primary driver of persistent hyperhomocysteinemia, combined oral supplementation with methylfolate (1,000 µg once daily) and vitamin B12 (cyanocobalamin, 1,000 µg once daily) was initiated. After approximately 1 month of therapy, plasma homocysteine decreased from 66 μmol/L to 38 μmol/L, representing a reduction of about 40%–45%. With continued treatment, a subsequent measurement demonstrated a further decline to 24.6 μmol/L after 8 weeks, indicating a sustained and clinically meaningful improvement in homocysteine concentrations. Throughout this period, methylmalonic acid and other available biochemical markers, including acylcarnitine profile, free and total carnitine levels, and urine organic acids assay, remained within reference ranges, supporting the interpretation that the response reflected enhanced one-carbon remethylation capacity rather than correction of overt cobalamin deficiency.

### Investigations

Serial biochemical testing over 3 years demonstrated persistent moderate to intermediate elevation of plasma total homocysteine (34–65 μmol/L, most values 50–60 μmol/L). Plasma methionine was generally within the normal range with intermittent mild elevations, while cystathionine, methylmalonic acid, and folate remained normal. Red cell folate became elevated during supplementation, and serum vitamin B12 reached supraphysiologic levels during intensive hydroxocobalamin therapy. Longitudinal biochemical results, including homocysteine, methionine, vitamin B12, and red-cell folate in relation to each therapeutic phase, are summarized in Table 1.

**TABLE 1:** Longitudinal biochemical profile and therapeutic interventions in an adolescent male with persistent isolated hyperhomocysteinemia.

Time period/intervention	Homocysteine (≤12.0 umol/L) + MMA (<0.47 umol/L)	Methionine (13 - 44 umol/L)	*Red-cell folate (1,187 - 2,854 nmol/L)	Vitamin B12 (145–569 pmol/L)	**Comment
Pre-B6 phase	63.5 (H); 53.2 (H)	–	–	–	Initial detection of marked hyperhomocysteinemia, repeated on two occasions
B6 phase 500mg/day (3 months)	59.6 (H); 52.9 (H); 65.3 (H)	18; 30	*574	282; 462	No clear reduction in homocysteine despite B6, arguing against primary transsulfuration defect
Early hydroxocobalamin phase (5mg/day)	50.0 (H)	–	–	>4,427 (H)	Start of high-dose (5mg) parenteral B12, homocysteine still elevated
Peak early response on hydroxocobalamin (5mg/day)	34.2 (H)	52 (H)	–	>4,427 (H)	Transient decline in homocysteine, with intermittent high methionine
Continued hydroxocobalamin therapy (14 months) (10mg/day)	40.4 (H); 39.7 (H); 49.1 (H); 58.6 (H); 50.5 (H)	38; 36; 24; 14; 37	–	>4,427 (H); 3,318 (H); 1,635 (H)	Persistent intermediate-range hyperhomocysteinemia despite supraphysiologic B12 levels
Hydroxocobalamin therapy + high-dose betaine (6g/day) (9 months)	63.7 (H)	17	–	>4,427 (H)	Homocysteine still markedly elevated at start of betaine
Ongoing betaine therapy (12g/day) (1 month)	55.3 (H); 58.8 (H); 46.0 (H) MMA 0.23	24; 55 (H); 51 (H)	1,590	1,327 (H); 794 (H)	Only modest, inconsistent improvement in homocysteine levels despite prolonged betaine treatment
Before methylfolate (1mg/day) + cyanocobalamin (1mg/day)	66.9 (H) MMA 0.13	39	1,465	672	Baseline value before initiation of treatment
4 weeks after methylfolate + Cyanocobalamin (1mg/day)	38.0 (H) MMA <0.05	19	2,246	1,355 (H)	Marked ∼42% reduction in homocysteine with combined methylfolate and cyanocobalamin
8 weeks after methylfolate + Cyanocobalamin (1mg/day)	24.6 MMA 0.27	-	2,292	1,956 (H)	Sustained improvement with homocysteine in the moderate range; complete blood count (CBC) normal

* Our lab changed the methodology and the reference ranges for Red Cell Folate over the years. This value was measured with an older methodology and with a reference range of 182–834 nmol/L. Plaese that reference ranges for B12 and homocysteine levels were also changed at that time.

** Cystathionine levels were repeatedly normal in multiple collections of plasma amino acid assay and hence are not reported in the table above.

Autoimmune and thrombophilia evaluation demonstrated low-titer antinuclear antibody and anticardiolipin IgG without clinical or laboratory evidence of systemic autoimmune disease or antiphospholipid syndrome.

Extensive metabolic and genetic investigations were performed. These included next-generation sequencing of genes associated with targets of newborn screening. Analysis restricted to genes for homocystinuria and methylmalonic aciduria (*CBS, AHCY, MAT1A, GNMT, SLC25A13, ADK, MTHFR, MTR, MTRR, MMADHC*), chromosomal microarray analysis, mitochondrial DNA sequencing, fibroblast respiratory chain enzyme activity testing, and cobalamin complementation studies in cultured fibroblasts. Clinical whole-exome sequencing (WES) and research long-read genome sequencing were also undertaken and did not identify any pathogenic variants consistent with a known inborn error of metabolism. The WES identified three heterozygous variants in MTFMT, RAG1 and RAG2, which were not considered related to the patient’s clinical presentation. Mitochondrial DNA sequencing revealed a heterozygous variant of uncertain significance in the TK2 gene (c.122C>A, p. (Pro41His)), which was also deemed not clinically significant to the presentation.

**FIGURE 1:**
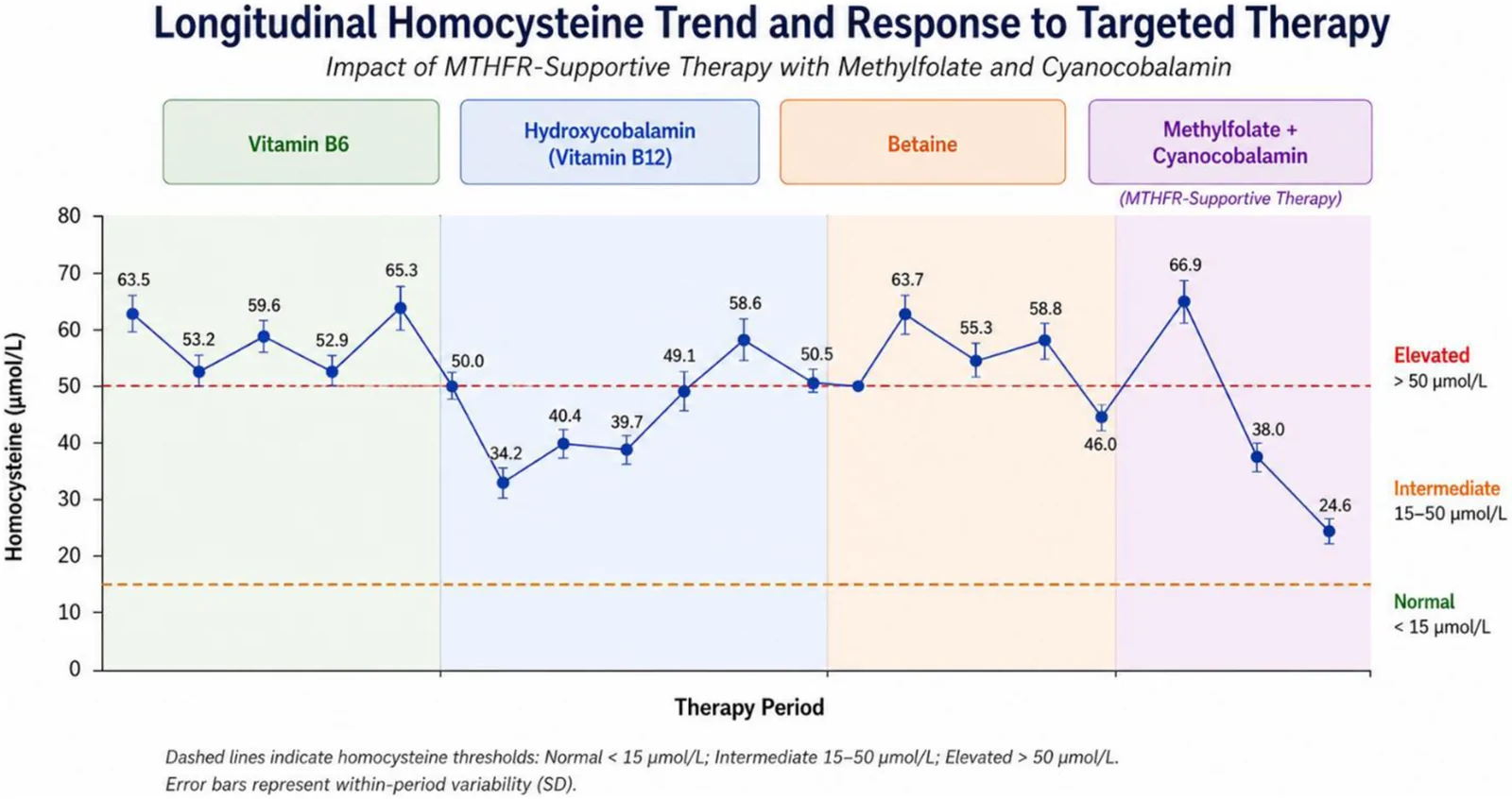
Longitudinal plasma homocysteine levels with sequential treatment phases. Figure 1 shows plasma total homocysteine (µmol/L) across the main therapeutic phases. Individual measurements are plotted as circles and connected by a line to illustrate the trajectory over time. The shaded regions represents the normal reference range for adolescents (≤15.0 umol/L). The patient shows persistent intermediate-range hyperhomocysteinemia (approximately 50–65 μmol/L) during pyridoxine, high-dose hydroxocobalamin, and high-dose betaine therapy. Following the introduction of combined oral methylfolate (1,000 µg/day) and vitamin B12 (1,000 µg/day), homocysteine decreased from 66.9 to 38.0 μmol/L after approximately 1 month, and further to 24.6 μmol/L on continued treatment after 2 months.

Re-analysis of the clinical exome data identified homozygosity for the common polymorphism MTHFR c.665C>T (p.Ala222Val) variant. Family testing confirmed the inheritance pattern: the father was also homozygous TT with plasma total homocysteine 10.9 μmol/L (normal range <15 μmol/L), while the mother was heterozygous CT with plasma total homocysteine 10.4 μmol/L (normal). No other pathogenic or likely pathogenic variants consistent with a classical inborn error of sulfur amino acid metabolism were identified. These findings excluded classical homocystinuria and inherited cobalamin remethylation disorders and supported the interpretation of multifactorial isolated hyperhomocysteinemia. The time course of plasma total homocysteine in relation to sequential cofactor therapies is illustrated in Figure 1.

## Discussion

In this adolescent with persistent isolated hyperhomocysteinemia, extensive biochemical testing and genetic testing excluded classical inborn errors of sulfur amino acid metabolism or other genetically inherited causes. The remaining clinical picture was that of a multifactorial disturbance of one-carbon metabolism shaped by celiac disease that likely led to micronutrient vulnerability andhomozygous *MTHFR* c.665C>T polymorphism.

Celiac disease is a recognized risk factor for disturbances in B-vitamin status and homocysteine metabolism, even in patients on a strict gluten-free diet (Saibeni et al., 2005; Hadithi et al., 2009). Gluten-free products are often low in folate and vitamin B12, and cross-sectional studies have demonstrated lower folate intake, lower serum folate, and higher homocysteine in treated celiac patients compared with controls (Saibeni et al., 2005; Hadithi et al., 2009). Hyperhomocysteinemia in this context is typically moderate or intermediate in severity (15–100 μmol/L) but has been associated with increased risk of venous thromboembolism, particularly when elevations are long-standing (Den Heijer et al., 2005; Coppola et al., 2000). Our patient’s persistent intermediate-range homocysteine (50–65 μmol/L) falls into this risk category.

A further challenge is that nutritional deficiency may be functional rather than overt. Serum folate and B12 can remain within reference ranges despite reduced tissue availability. Elevated homocysteine and methylmalonic acid are more sensitive indicators of functional B-vitamin deficiency than serum vitamin levels alone (Stabler, 2013). In our patient, serum vitamin B12 was in the mid-normal range at baseline (282–496 pmol/L) and became repeatedly supraphysiologic during supplementation (frequently >4,427 pmol/L and remaining ≥672–3,318 pmol/L thereafter), while red cell folate was normal or high (574 nmol/L, rising to 1,465–2,292 nmol/L); despite this, plasma homocysteine remained elevated (24.6 μmol/L at last measurement), consistent with incomplete correction of intracellular one-carbon metabolism.

Homozygosity for the *MTHFR* c.665C>T polymorphism represents an additional, genetically determined vulnerability. The TT genotype reduces *MTHFR* activity, leading to lower 5-methyltetrahydrofolate and higher homocysteine, particularly when folate status is marginal (Graydon et al., 2019; Huang et al., 2018). Large studies have shown that individuals with the TT genotype require higher folate levels to normalize homocysteine compared with CC or CT genotypes (Graydon et al., 2019; Huang et al., 2018). In this context, celiac disease could reduce folate reserve, while *MTHFR* 665TT lowered the metabolic threshold for homocysteine accumulation, together sustaining persistent hyperhomocysteinemia. Family studies strengthened this hypothesis. The patient’s father, who is also homozygous for *MTHFR* c.665C>T (TT), had a normal plasma homocysteine level (10.9 μmol/L), and the mother, a heterozygous carrier (CT), likewise had a normal homocysteine level (10.4 μmol/L). This pattern is consistent with population data showing that TT homozygosity is common and typically confers only a modest average increase in homocysteine (≈20–25%) rather than a deterministic hyperhomocysteinemic phenotype. The absence of biochemical abnormality in the TT father underscores that *MTHFR* c.665C>T functions as a susceptibility factor whose phenotypic expression depends on additional stressors, rather than as a monogenic cause of disease. In our patient, celiac-related impairment of one-carbon reserve appears to have acted synergistically with the TT genotype to sustain intermediate-range hyperhomocysteinemia, whereas the same genotype in the father, in the absence of celiac disease, remained clinically silent. (Graydon et al., 2019; Huang et al., 2018).

The patient’s therapeutic course provides insight into underlying pathophysiology. Pyridoxine supplementation, aimed at enhancing transsulfuration via vitamin B6-dependent cystathionine β-synthase, did not change homocysteine levels, and normal cystathionine argues against a primary transsulfuration defect. Intensive hydroxocobalamin treatment led to transient improvement but failed to produce sustained normalization despite very high serum B12. Because hydroxocobalamin is converted *in vivo* to methylcobalamin, this pattern suggests that cobalamin delivery alone could not overcome limitation in upstream folate-dependent generation of 5-methyltetrahydrofolate in the setting of *MTHFR* 665TT and celiac disease (Froese et al., 2019; Stabler, 2013; Graydon et al., 2019; Huang et al., 2018). High-dose betaine resulted in only modest reductions in plasma homocysteine. As betaine remethylates homocysteine via hepatic betaine-homocysteine methyltransferase (BHMT) in a pathway that is independent of folate and vitamin B12, this limited response may reflect the relatively minor contribution of BHMT to total remethylation capacity in this patient and the overall severity of the underlying disturbance in one-carbon flux. (Choi and Friso, 2023).

Based on these observations, we introduced combined oral methylfolate (1,000 µg once daily) and vitamin B12 (cyanocobalamin 1,000 µg once daily) to support the folate-dependent remethylation pathway more directly. After approximately 1 month of therapy, plasma homocysteine decreased from 66 to 38 μmol/L, representing a reduction of about 40%–45%. With continued treatment, a subsequent measurement demonstrated a further decline to 24.6 μmol/L, indicating a sustained and clinically meaningful improvement in homocysteine concentrations after 8 weeks. Throughout this period, methylmalonic acid and other available biochemical markers remained within reference ranges, supporting the interpretation that the response reflected enhanced one-carbon remethylation capacity rather than correction of overt cobalamin deficiency. This pattern is consistent with emerging adult data showing that methylfolate-containing regimens, usually in combination with vitamin B12 and vitamin B6, can substantially reduce homocysteine in individuals with *MTHFR* polymorphisms, although standardized pediatric dosing protocols have not yet been established and there are limitations to extrapolating adult data to this adolescent case (Pokushalov et al., 2024).

One limitation of this work is that we did not measure 5-MTHF, which would have provided additional biochemical support for the proposed mechanism. Another limitation is that we do not have longer-term data beyond 8 weeks of treatment, which could have strengthened our therapeutic conclusion.

From a broader perspective, this case adds to the literature linking *MTHFR* variants to diverse phenotypes, including vascular risk and illustrates interaction with celiac-related nutritional vulnerabilities (Graydon et al., 2019; Huang et al., 2018; Saibeni et al., 2005; Hadithi et al., 2009; Uchida et al., 2014; Calderón-Larrañaga et al., 2020). This patient presented with type 1 diabetes, celiac disease, congenital unilateral sensorineural hearing loss, and homozygous polymorphism of *MTHFR* c.665C>T with persistent hyperhomocysteinemia. Associations between MTHFR variants and acquired hearing impairment have been described (Uchida et al., 2014; Hamidi et al., 2019); however, our patient had congenital unilateral sensorineural hearing loss, which may represent an independent comorbidity. An association between this polymorphism and diabetes mellitus has also been reported, although primarily in individuals with type 2 diabetes (Meng et al., 2019). While no causal relationship can be inferred, this constellation of findings underscores the value of considering shared genetic modifiers when investigating unexplained metabolic abnormalities.

Clinically, the key lesson is that persistent hyperhomocysteinemia in a patient with celiac disease is not necessarily a genetic or metabolic disease, but it is also not benign. Once inborn errors of metabolism are excluded, clinicians should actively evaluate for gene-nutrient interactions such as *MTHFR* polymorphisms, optimize dietary sources of folate and B vitamins, and consider targeted use of active cofactors (e.g., methylfolate and vitamin B12/Cyanocobalamin) (Saibeni et al., 2005; Hadithi et al., 2009; Pokushalov et al., 2024). Early recognition may prevent excessive diagnostic testing and allow timely, rational management.

## Conclusion

This case demonstrates that persistent hyperhomocysteinemia in adolescence may arise from complex interactions between common genetic variants and acquired nutritional conditions rather than from classical monogenic defects. In the context of celiac disease, even with strict dietary adherence, homozygous *MTHFR* c.665C>T can lower the threshold for metabolic decompensation and sustain intermediate range hyperhomocysteinemia; however, the normal homocysteine levels observed in the patient’s TT homozygous father highlight that this polymorphism is a modifier, not a stand-alone cause. Targeted supplementation with methylfolate and vitamin B12/cyanocobalamin provided a rational therapeutic strategy leading to a sustained reduction in homocysteine without evidence of overt cobalamin deficiency. Recognition of such multifactorial mechanisms and the interpretive value of family biochemistry is important for accurate diagnosis, appropriate counseling, and effective, pathway-directed management in patients with celiac disease and unexplained hyperhomocysteinemia.

Learning Points.

1. Persistent isolated hyperhomocysteinemia in adolescents may reflect a gene–nutrient interaction between *MTHFR* polymorphism and celiac disease, rather than a classical inborn error.
2. Combined methylfolate and vitamin B12 can be effective treatment in patients with persistent hyperhomocysteinemia of multifactorial origin when standard cofactor therapies have failed.

## Data Availability

The datasets presented in this article are not readily available because of ethical and privacy restrictions. Requests to access the datasets should be directed to the corresponding author.

## References

[B1] Calderón-Larrañaga A. Saadeh M. Hooshmand B. Refsum H. Smith A. D. Marengoni A. (2020). Association of homocysteine, methionine, and *MTHFR* 677C>T polymorphism with rate of cardiovascular multimorbidity development in older adults in Sweden. JAMA Netw. Open 3 (5). 10.1001/jamanetworkopen.2020.5316 32432712 PMC7240355

[B3] Choi S. W. Friso S. (2023). Modulation of DNA methylation by one-carbon metabolism: a milestone for healthy aging. Nutr. Res. Pract. 17 (4), 597–615. 10.4162/nrp.2023.17.4.597 37529262 PMC10375321

[B4] Coppola A. Davi G. De Stefano V. Mancini F. P. Cerbone A. M. Di Minno G. (2000). Homocysteine, coagulation, platelet function, and thrombosis. Semin. Thromb. Hemost. 26 (3), 243–254. 10.1055/s-2000-8469 11011842

[B5] Den Heijer M. Lewington S. Clarke R. (2005). Homocysteine, MTHFR and risk of venous thrombosis: a meta-analysis of published epidemiological studies. J. Thromb. Haemost. 3 (2), 292–299. 10.1111/j.1538-7836.2005.01141.x 15670035

[B6] Froese D. S. Fowler B. Baumgartner M. R. (2019). Vitamin B12, folate, and the methionine remethylation Cycle—Biochemistry, pathways, and regulation. J. Inherit. Metab. Dis. 42 (4), 673–685. 10.1002/jimd.12009 30693532

[B7] Graydon J. S. Claudio K. Baker S. Kocherla M. Ferreira M. Roche-Lima A. (2019). Ethnogeographic prevalence and implications of the 677C>T and 1298A>C *MTHFR* polymorphisms in US primary care populations. Biomark. Med. 13 (8), 649–661. 10.2217/bmm-2018-0392 31157538 PMC6630484

[B8] Hadithi M. Mulder C. J. Stam F. Azizi J. Crusius J. B. A. Peña A. S. (2009). Effect of B vitamin supplementation on plasma homocysteine levels in celiac disease. World J. Gastroenterol. 15 (8), 955–960. 10.3748/wjg.15.955 19248194 PMC2653396

[B9] Hamidi A. K. Yazdani N. Haj Seyedjavadi K. Ahrabi N. Z. Tajdini A. Aghazadeh K. (2019). Mthfr and ApoE genetic variants association with sudden sensorineural hearing loss. Am. J. Otolaryngology 40 (2), 260–264. 10.1016/j.amjoto.2018.10.015 30477909

[B10] Huang X. Qin X. Yang W. Liu L. Jiang C. Zhang X. (2018). *MTHFR* gene and serum folate interaction on serum homocysteine lowering: prospect for precision folic acid treatment. Arterioscler. Thromb. Vasc. Biol. 38 (3), 679–685. 10.1161/ATVBAHA.117.310211 29371246

[B11] Meng Y. Liu X. Ma K. Zhang L. Lu M. Zhao M. (2019). Association of MTHFR C677T polymorphism and type 2 diabetes mellitus (T2DM) susceptibility. Mol. Genet. Genomic Med. 7, e1020. 10.1002/mgg3.1020 31663297 PMC6900375

[B12] Morris A. A. Kožich V. Santra S. Andria G. Ben-Omran T. I. M. Chakrapani A. B. (2017). Guidelines for the diagnosis and management of cystathionine beta-synthase deficiency. J. Inherit. Metab. Dis. 40 (1), 49–74. 10.1007/s10545-016-9979-0 27778219 PMC5203861

[B13] Pokushalov E. Ponomarenko A. Bayramova S. Garcia C. Pak I. Shrainer E. (2024). Effect of methylfolate, Pyridoxal-5'-Phosphate, and methylcobalamin (Soloways^TM^) supplementation on homocysteine and low-density lipoprotein cholesterol levels in patients with methylenetetrahydrofolate reductase, methionine synthase, and methionine synthase reductase polymorphisms: a randomized controlled trial. Nutrients 16 (11), 1550. Published 2024 May 21. 10.3390/nu16111550 38892484 PMC11173557

[B14] Saibeni S. Lecchi A. Meucci G. Cattaneo M. Tagliabue L. Rondonotti E. (2005). Prevalence of hyperhomocysteinemia in adult gluten-sensitive enteropathy at diagnosis: role of B12, folate, and genetics. Clin. Gastroenterol. Hepatol. 3 (6), 574–580. 10.1016/s1542-3565(05)00022-4 15952099

[B15] Stabler S. P. (2013). Vitamin B12 deficiency. N. Engl. J. Med. 368 (2), 149–160. 10.1056/nejmcp1113996 23301732

[B16] Uchida Y. Sugiura S. Ueda H. Nakashima T. Ando F. Shimokata H. (2014). The association between hearing impairment and polymorphisms of genes encoding inflammatory mediators in Japanese aged population. Immun Ageing 11 (8). 10.1186/s12979-014-0018-4 25469152 PMC4252019

